# Expressions of miR-21 and miR-210 in Breast Cancer and Their Predictive Values for Prognosis

**Published:** 2020-01

**Authors:** Xiaofei WU

**Affiliations:** Department of Neurology, Wuhan General Hospital of Guangzhou Military, Wuhan 430070, P.R. China

**Keywords:** miR-21, miR-210, Breast cancer, Prognosis, Clinicopathology

## Abstract

**Background:**

We aimed to investigate the expressions of miR-21 and miR-210 in the breast cancer tissue and their correlation with clinicopathological features and prognosis.

**Methods:**

A retrospective analysis was performed on 68 patients with breast cancer treated surgically in Wuhan General Hospital of Guangzhou Military in 2014–2015. The breast cancer tissue and the adjacent normal tissue were collected from the patients. Quantitative real-time PCR (qRT-PCR) was used to detect the expression levels of miR-21 and miR-210 in the breast cancer and adjacent normal tissues.

**Results:**

According to qRT-PCR, the expression levels of miR-210 and miR-21 in the breast cancer tissue were significantly higher than those in the adjacent normal tissue (*P*<0.05), which were significantly correlated with lymph node metastasis, clinical staging and differentiation of patients (*P*<0.05). miR-21 and miR-210 were significantly positive correlated in both breast cancer tissues and adjacent normal tissues (r=0.7014, 0.7502, *P*<0.001). The survival rate in the miR-210 high expression group was significantly lower than that in the miR-210 low expression group (*P*<0.05), whereas there was no significant difference between the miR-21 high and low expression groups.

**Conclusion:**

miR-21 and miR-210 are highly expressed in the breast cancer tissue and significantly correlated with lymph node metastasis, clinical staging and differentiation. miR-210, the up-regulated expression of which is related to the poor prognosis of patients with breast cancer, may be a potential prognostic indicator for breast cancer, which can be used to judge the prognosis.

## Introduction

As a malignant tumor in women with the highest incidence rate, breast cancer accounts for one quarter of female cancers, with a mortality rate accounting for 15% (1). The disease can be divided into different molecular subtypes according to progesterone receptor, estrogen receptor, etc. (2). The prognosis of the patients has been improved in recent years but remains unsatisfactory, so it is urgent to find new treatments of the disease. With the deepening of research, recent studies have found that miRNA regulates the early development of cells and is involved in their differentiation, development and apoptosis (3, 4), which is also involved in the occurrence and development of tumors through regulating the function of its target genes (5).

As a miRNA used for diagnostic research, miR-21 functions as a proto-oncogene (6), and is highly expressed in the occurrence and development of tumors (7). Studies have shown that it is carcinogenic in gastric, liver and colon cancers (8–10). miR-21 functions as an oncogene and biomarker for breast cancer (11), and its expression level is significantly up-regulated in the breast cancer tissue (12). miR-210 is an endogenous non-coding RNA, the expression level of which is up-regulated in hypoxia environment in vitro experiments (13). According to a recent study, it is up- or down-regulated in tumors, thereby functioning as a tumor suppressor gene or oncogene (14). miR-210 is a marker for distinguishing acute lymphoblastic leukemia from acute myeloblastic leukemia, correlated with acute lymphoblastic leukemia in children (15, 16). Therefore, the roles of miR-21 and miR-210 are different, depending on the type of cancers.

There are currently few reports on miR-21 and miR-210 in breast cancer, and there is no research report on whether there is significant correlation between miR-21 and miR-210. Therefore, miR-21 and miR-210 in the breast cancer tissue were explored in this paper in terms of their expression levels, their correlation with clinicopathological features, the correlation between the two and their effects on the prognosis.

## Materials and Methods

### General information

A total of 68 patients with breast cancer treated surgically in Wuhan General Hospital of Guangzhou Military, China from January 2014 to May 2015 were enrolled in this study, aged 30–67 yr old with an average age of 42.77±8.62 yr old. The breast cancer and adjacent normal tissues from the patients were obtained for experiments. There were 22 cases in clinical stage I, 21 cases in stage II, and 25 cases in stage III. General information is shown in Table 1. Inclusion criteria: Patients pathologically diagnosed with breast cancer who had undergone surgical treatment were included. Exclusion criteria: Patients who had received radiotherapy and chemotherapy before specimens were taken were excluded; patients with other severe organ diseases; patients who did not cooperate with examination; patients with cognitive and communication disorders.

**Table 1:** General information (n[%])

***Factors***	***n=68***
Age (yr)	
≤40	30 (44.12)
>40	38 (55.88)
Body weight (kg)	
≤60	20 (29.41)
>60	48 (70.59)
Height (cm)	
≤160	31 (45.59)
>160	37 (54.41)
Smoking	
Yes	22 (32.35)
No	46 (67.65)
Alcoholism	
Yes	27 (39.71)
No	41 (60.29)
Clinical staging	
Stage I	22 (32.35)
Stage II	21 (30.88)
Stage III	25 (36.77)
Lymph node metastasis	
Yes	37 (54.41)
No	31 (45.59)
Differentiation	
Poorly differentiated	30 (44.12)
Highly differentiated	38 (55.88)

The breast cancer tissue and the normal tissue without cancer cell infiltration under the microscope which was over 2 cm from the tumor margin were immediately taken after operation and stored in a refrigerator at −80 °C. All patients cooperated with medical staff to complete relevant diagnosis and treatment. Patients and their families were informed in advance before the study and signed an informed consent form.

### Main reagents and instruments

Reverse transcription kit Fermentas was purchased from Beijing Think-Far Technology Co., Ltd., qRT-PCR kit from Thermo Fisher Scientific, China, Trizol kit from Shanghai Pufei Biotech Co., Ltd., UV-3100PC spectrophotometer from Shanghai Mapada Instruments Co., Ltd.

### Experimental methods

The total RNA in the breast cancer and adjacent normal tissues was extracted with one-step extraction method using the Trizol kit, with specific steps carried out in strict accordance with the instructions. The UV-3100PC spectrophotometer was used to detect the concentration and purity, 1% denatured agarose gel electrophoresis to detect the integrity. The RNA extracted was reversely transcribed into cDNA as a template for experiments. Primer sequences were designed and synthesized by Sangon Biotech (Shanghai) Co., Ltd., and U6 was used as an internal reference gene in this experiment. The reverse transcription system was 15 μL in total: 0.5 μL of primers for reverse transcription, 0.5 μL of reverse transcriptase, 1.5 μL of buffer, 2 μL of RNA, DEPC-treated water used to complement to 15 μL. Reaction conditions were at 37 °C for 10 min and at 95 °C for 5 min. The synthesized cDNA was stored at 4 °C.

The quantitative real-time PCR (qRT-PCR) reaction system was prepared based on the instructions. The system was 20 μL in total: 10 μL of 2×TaqPCR Master Mix, 1 μL of primers, 1.33 μL of cDNA (diluted at 1: 15), 10 μL of TaqMan 2× Universal PCR Master Mix II, DEPC-treated water used to complement to 20 μL. qRT-PCR instrument was used for PCR amplification, and reaction conditions were as follows: predenaturation at 95 °C for 10 min, at 95 °C for 15 s, at 65 °C for 30 s and at 72 °C for 30 s, for 40 cycles. PCR products were stored at 4 °C. There were 3 same samples in each group, and 2^–ΔCT^ was used to analyze the expression levels of miR-21 and miR-210. The primer sequences are shown in Table 2.

**Table 2:** miR-21 and miR-210 primer sequences

***Primer sequences***	***Upstream primer***	***Downstream primer***
miR-21	5′- GCTGGCGACGGGACATTATTAC-3′	5′- AGGGCTATGCCGCCTAAGTACG -3′
miR-210	5′- GCTGTGCGTGTGACAGC-3′	5′- GTGCAGGGTCCGAGGT-3′
U6	5′-GCTTCGGCAGCACATATACTAAAAT-3′	5′-CGCTTCACGAATTTGCGTGTCAT-3′

### Observation indicators

The relationship between miR-21 and miR-210 in breast cancer patients and adjacent normal tissues was observed, and the relationship between miR-21 and miR-210 and clinicopathological features were explored. The patients were followed up for three years for their survival situation. The relationship between miR-21, miR-210 expression and patient survival rate were analyzed; the correlation between miR-21 and miR-210 in adjacent tissues and cancer tissues was analyzed.

### Statistical methods

SPSS20.0 software package [Bizinsight (Beijing) Information Technology Co., Ltd.] was used to statistically analyze the data, GraphPad Prism 7 to plot figures. Measurement data were expressed as mean±standard deviation, and t test was used for comparison between the two groups. Count data were tested by chi-square. Pearson correlation coefficient was used for data confirming to bivariate normal distribution, Kaplan-Meier for survival analysis, Log-rank for test. *P* <0.05 indicates a statistically significant difference.

## Results

### Expressions of miR-21 and miR-210

The expression of miR-21 in the breast cancer tissue was (4.77±1.36), significantly higher than (4.17±1.01) in the adjacent normal tissue (*t*= 2.921, *P*=0.004). The expression of miR-210 in the breast cancer tissue was (1.16±0.46), significantly higher than (0.85±0.27) in the adjacent normal tissue (*t* = 4.793, *P*<0.001) (Table 3).

**Table 3:** Expressions of miR-21 and miR-210 (x̅±sd)

***Groups***	***Cancer tissue (n=68)***	***adjacent tissue (n=68)***	**t**	**P**
miR-21	4.77±1.36	4.17±1.01	2.921	0.004
miR-210	1.16±0.46	0.85±0.27	4.793	<0.001

### Correlation of miR-21 expression with clinicopathological features

The expression of miR-21 in patients with breast cancer was not significantly correlated with age, body weight and height, but significantly correlated with lymph node metastasis, clinical staging and differentiation (*P*<0.05) (Table 4).

**Table 4:** Correlation of miR-21 expression with clinicopathological features (x̅±sd)

***Groups***	***n (n=68)***	***miR-21***	***t/F***	**P**
Age (yr)			0.353	0.725
≤40	30	4.71±1.44		
>40	38	4.83±1.35		
Body weight (kg)			0.050	0.957
≤60	20	4.76±1.31		
>60	48	4.78±1.41		
Height (cm)			0.246	0.807
≤160	31	4.81±1.36		
>160	37	4.73±1.32		
Clinical staging			10.710	<0.001
Stage I	22	4.01±1.31		
Stage II	21	4.52±1.42		
Stage III	25	5.78±1.34		
Lymph node metastasis			2.130	0.037
Yes	37	5.13±1.37		
No	31	4.41±1.41		
Differentiation			2.339	0.022
Poorly differentiated	30	5.18±1.39		
Highly differentiated	38	4.36±1.47		

### Correlation of miR-210 expression with clinicopathological features

The expression of miR-210 in patients with breast cancer was not significantly correlated with age, body weight and height, but significantly correlated with lymph node metastasis, clinical staging and differentiation (*P*<0.05) (Table 5).

**Table 5:** Correlation of miR-210 expression with clinicopathological features (x̅±sd)

***Groups***	***n (n=68)***	***miR-210***	***t/F***	**P**
Age (yr)			1.420	0.160
≤40	30	1.11±0.31		
>40	38	1.21±0.27		
Body weight (kg)			1.094	0.278
≤60	20	1.12±0.29		
>60	48	1.18±0.16		
Height (cm)			0.950	0.345
≤160	31	1.19±0.27		
>160	37	1.13±0.25		
Clinical staging			72.01	<0.001
Stage I	22	0.84±0.21		
Stage II	21	1.04±0.18		
Stage III	25	1.60±0.27		
Lymph node metastasis			4.124	0.001
Yes	37	1.29±0.24		
No	31	1.03±0.28		
Differentiation			2.395	0.019
Poorly differentiated	30	1.24±0.29		
Highly differentiated	38	1.08±0.26		

### Correlation of miR-21 expression with miR-210 expression

**Fig. 1:**
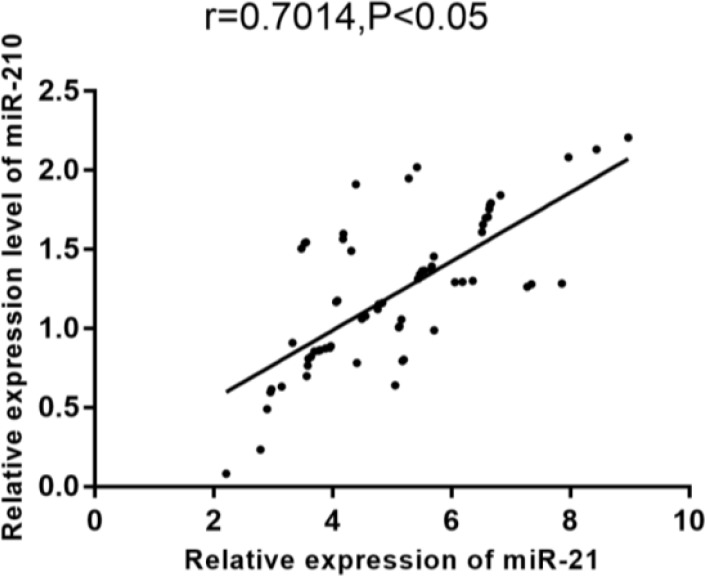
Correlation of miR-21 expression with miR-210 expression According to Pearson correlation coefficient, miR-21 expression was positively correlated with miR-210 expression in the breast cancer tissue (r=0.7014, *P* <0.05)

**Fig. 2:**
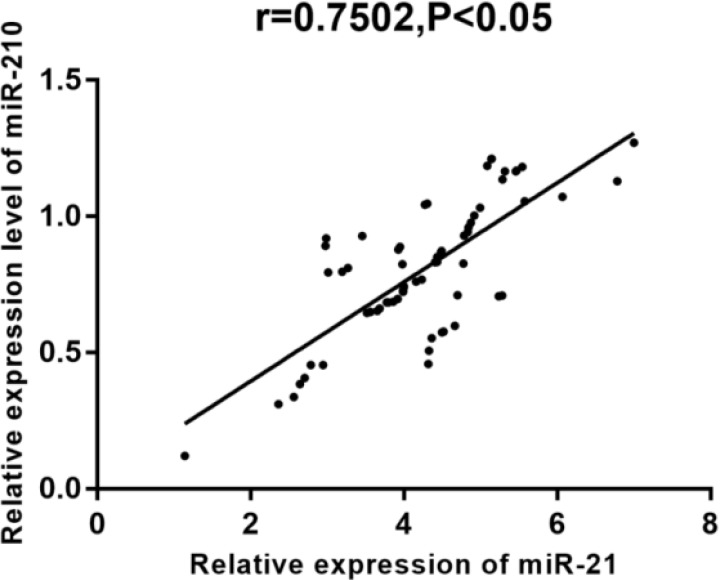
Expression correlation of miR-21 and miR-210 in adjacent normal tissues. Pearson correlation coefficient results showed that the expression levels of miR-21 and miR-210 were significantly positively correlated with adjacent normal tissues (r=0.7502, *P*<0.05)

According to Pearson correlation coefficient, miR-21 expression was positively correlated with miR-210 expression in both the breast cancer tissue and adjacent normal tissue (r = 0.7014, 0.7502, *P* <0.001) (Fig. 1–2).

### Correlation of miR-21 and miR-210 expressions with prognosis

**Fig. 3:**
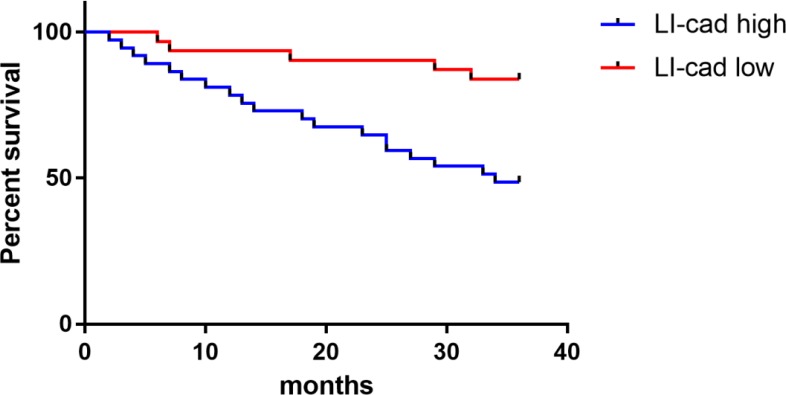
Correlation of miR-210 expression with survival. Up to the end of follow-up, the survival rate was 48.65% in the miR-210 high expression group, significantly lower than 83.87% in the miR-210 low expression group (*P* <0.05)

The survival data of the breast cancer patients were counted, and the average value (1.04) of miR-210 expression was used as a boundary. Of 68 cases, 31 with a miR-210 value less than 1.04 were in the low expression group, another 37 with a miR-210 value greater than or equal to 1.04 in the high expression group. The survival rate was 48.65% in the high expression group and 83.87% in the low expression group, with follow-up time up to June 1, 2018. As shown in Fig. 3, the survival rate in the miR-210 low expression group was significantly higher than that in the miR-210 high expression group (*P*<0.05).

### Correlation of miR-21 expression with prognosis

**Fig. 4:**
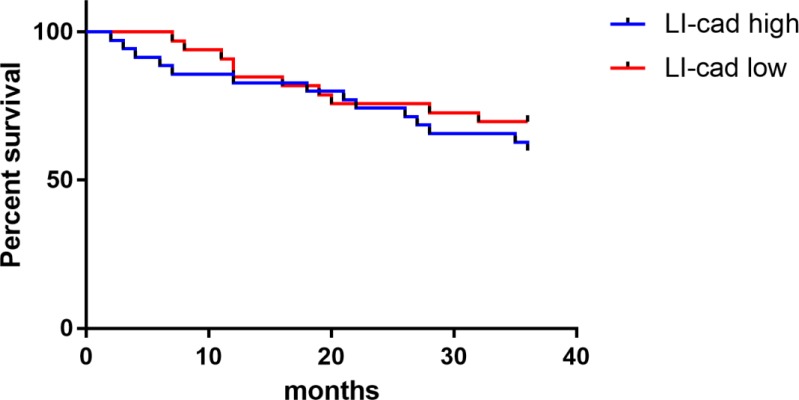
Correlation of miR-21 expression with survival.Up to the end of follow-up, the survival rate was 60.00% in the miR-21 high expression group, lower than 69.70% in the miR-21 low expression group (*P* > 0.05)

The survival data of breast cancer patients were counted, and the average value (4.24) of miR-21 expression was used as a boundary. Of 68 cases, 33 with a miR-21 value less than 4.24 were in the low expression group, another 35 with a miR-21 value greater than or equal to 4.24 in the high expression group. The survival rate was 60.00% in the high expression group and 69.70% in the low expression group, with follow-up time up to June 1, 2018. As shown in Fig. 4, there was no significant difference in the survival rate between the miR-21 high and low expression groups.

## Discussion

Breast cancer is a common malignant tumor in women (17), and its mode of onset is mainly abnormal proliferation of cancer cells to destroy the normal tissue and change the structure of normal breast. Gene mutation occurs in breast epithelial cells after the cells are stimulated and then causes abnormal proliferation and disordered malignant proliferation (18).

Breast cancer is more common in people aged 40–60 years old, with a high incidence rate in women before and after menopause. Breast cancer is mainly caused by heredity, eating disorders and radiation, the incidence rate of which has been increasing with the development of electronic industry and changes of living standards. The disease is clinically manifested as breast mass, axillary lymph node enlargement and breast pain, the mortality rate of which is high due to its unapparent symptoms and patients’ little attention to the condition (19–21).

Early diagnosis and treatment are important for the prognosis of breast cancer, and miRNA can be used as a diagnostic marker because it is specifically expressed in the diseased tissue and serum. miRNA is specific in the tissue and plasma of patients with tumors, indicating that miRNA can be used as a new tumor marker (22). According to a study (23), miR-21, the expression of which is up-regulated in solid tumors, is involved in the regulation of tumor-inhibiting factors and related to the growth, invasion and metastasis of tumor cells, suggesting that miR-21 and miR-210 predict the survival rate of patients with breast cancer. In a study, miR-210 content is high in the serum of patients with breast cancer (23). However, there are currently few studies on miR-21 and miR-210 in the prognosis of the disease. Therefore, miR-21 and miR-210 in the breast cancer tissue were explored in this study in terms of their expression levels, their correlation with clinicopathological features, the correlation between the two and their effects on the prognosis. In this study, the expression of miR-21 and miR-210 in the breast cancer tissue was significantly higher than that in the adjacent normal tissue, demonstrating that miR-21 and miR-210 are highly expressed in breast cancer. According to studies (24–26), miR-21 expression in the breast cancer tissue or serum is higher than that in the normal tissue or serum, suggesting that miR-21 functions as an oncogene and is closely related to the occurrence, development and metastasis of breast cancer. A study has shown that miR-210 is highly expressed in breast cancer and involved in the occurrence and development of tumors (23), which is similar to the results of this study.

In this study, the expressions of miR-21 and miR-210 in patients with breast cancer were significantly correlated with lymph node metastasis, clinical staging and differentiation (*P* <0.05). Moreover, miR-21 and miR-210 were gradually up-regulated with the severity of the disease. It is suggested that miR-21 and miR-210 have diagnostic value in breast cancer, similar to another study (27). miR-210 expression in colon cancer serum was closely related with tumor size, degree of invasion, lymph node metastasis and clinical stage (28). According to partial correlation analysis, miR-21 expression was positively correlated with miR-210 expression in both the breast cancer tissue and adjacent normal tissue. This indicated that the up-regulation of miR-21 expression is closely related to the expression of miR-210.

At present, there is no research on the correlation of miR-21 with miR-210 in breast cancer. So further studies are needed. According to the correlation of miR-21 and miR-210 expressions with the prognosis, the 3-year survival rate in the miR-210 high expression group was significantly lower than that in the miR-210 low expression group (*P* <0.05), whereas there was no significant difference between the miR-21 high and low expression groups (*P* > 0.05), indicating that miR-210 can be used as a biomarker for predicting the prognosis of breast cancer patients. Different miRNAs have different expressions in the same tumor, so further research is needed on this direction. High expression of miR-210 was an independent factor, and low expression of miR-210 showed better disease-free survival and overall survival than high expression (29). This is consistent with the results of this study. Studies have shown that miR-21 expression is significantly elevated to predict poor overall survival (*P* <0.05) (30), which is inconsistent with the results of this study, probably because the sample size selected in this study is small, the results have a certain bias.

In recent years, miR-21 and miR-210 are hot topics in clinical research. The expression and prognostic values of them in patients with breast cancer were comprehensively explored in this paper, which is hoped to provide references for clinical research. However, there are still some limitations. The sample size of this paper is small, the upstream and downstream regulatory genes and specific mechanisms of miR-21 and miR-210 have not been studied. In the future research, the prediction of treatment outcomes by different distribution on molecular subtypes of the two RNAs can be further explored, in order to help more correctly judge the prognosis.

## Conclusion

The study of miR-21 and miR-210 expressions is helpful to know the occurrence, development and biological behavior of breast cancer. miR-21 and miR-210 expressions are significantly correlated with lymph node metastasis, clinical staging and differentiation, and miR-210 can be used as a biomarker for the prognosis of breast cancer.

## Ethical considerations

Ethical issues (Including plagiarism, informed consent, misconduct, data fabrication and/or falsification, double publication and/or submission, redundancy, etc.) have been completely observed by the authors.
